# Inhibition and degradation of NRAS with a pan-NRAS monobody

**DOI:** 10.1038/s41388-024-03186-y

**Published:** 2024-10-08

**Authors:** Michael Whaby, Gayatri Ketavarapu, Akiko Koide, Megan Mazzei, Mubashir Mintoo, Eliezra Glasser, Unnatiben Patel, Cecile Nasarre, Matthew J. Sale, Frank McCormick, Shohei Koide, John P. O’Bryan

**Affiliations:** 1 Department of Cell and Molecular Pharmacology & Experimental Therapeutics, Medical University of South Carolina, Charleston, SC USA; 2 Hollings Cancer Center, Medical University of South Carolina, Charleston, SC USA; 3 Perlmutter Cancer Center, New York University Langone Health, New York, NY USA; 4 Department of Medicine, New York University School of Medicine, New York, NY USA; 5 Department of Biochemistry and Molecular Biology, Medical University of South Carolina, Charleston, SC USA; 6 Helen Diller Family Comprehensive Cancer Center, University of California San Francisco, San Francisco, CA USA; 7 Department of Cellular and Molecular Pharmacology, University of California San Francisco, San Francisco, CA USA; 8 Department of Biochemistry and Molecular Pharmacology, New York University School of Medicine, New York, NY USA; 9 Ralph H. Johnson VA Medical Center, Charleston, SC USA

**Keywords:** Skin cancer, Molecular biology

## Abstract

The RAS family GTPases are the most frequently mutated oncogene family in human cancers. Activating mutations in either of the three RAS isoforms (HRAS, KRAS, or NRAS) are found in nearly 20% of all human tumors with NRAS mutated in ~25% of melanomas. Despite remarkable advancements in therapies targeted against mutant KRAS, NRAS-specific pharmacologics are lacking. Thus, development of inhibitors of NRAS would address a critical unmet need to treating primary tumors harboring NRAS mutations as well as BRAF-mutant melanomas, which frequently develop resistance to clinically approved BRAF inhibitors through NRAS mutation. Building upon our previous studies with the monobody NS1 that recognizes HRAS and KRAS but not NRAS, here we report the development of a monobody that specifically binds to both GDP and GTP-bound states of NRAS and inhibits NRAS-mediated signaling in a mutation-agnostic manner. Further, this monobody can be formatted into a genetically encoded NRAS-specific degrader. Our study highlights the feasibility of developing NRAS selective inhibitors for therapeutic efforts.

## Introduction

The RAS GTPases are the most frequently mutated oncogene family in human cancers with RAS isoforms (HRAS, KRAS, and NRAS) regulating critical intracellular signaling cascades that control cell proliferation and survival [1]. Activating mutations in RAS occur in ~20% of all cancers with the majority of these mutations occurring in KRAS. However, NRAS is mutated in ~25% of melanomas, 20% of multiple myeloma, and more rarely in colorectal cancer (7.5%) [2, 3]. In contrast to KRAS mutations which are most frequently in codon 12, the most common NRAS mutations in melanomas occur at codon 61 (Q61R or Q61K). In addition, NRAS mutations frequently arise in BRAF-mutant melanomas as a mechanism of resistance in response to pharmacological inhibition of BRAF [4–7]. Although NRAS-mutant melanomas represent the most aggressive and second most common melanoma subtype behind BRAF mutant melanomas [4], there remain no available NRAS-targeted therapies. Thus, there is a critical need to develop inhibitors of NRAS that ideally target multiple NRAS mutants.

RAS has been a long sought-after target for cancer therapeutics [8]. Decades of efforts to develop RAS inhibitors passed with little promise due to the lack of recognizable binding pockets for small molecule inhibitors outside of the nucleotide-binding pocket [9, 10]. However, targeting nucleotide binding has not been viewed as feasible due to the high cellular concentration of guanine nucleotides coupled with the picomolar affinity of RAS for nucleotides, although recent studies challenge this notion [9–11]. Recently, the field has seen major advancements in the development of anti-RAS therapeutics. The FDA approval of two KRAS^G12C^ inhibitors, sotorasib and adagrasib, for use in patients with KRAS^G12C^-mutant non-small cell lung cancers has dispelled the premise that RAS is undruggable [12, 13]. Following on the success with these targeted compounds, an inhibitor of the most common KRAS mutant, KRAS^G12D^, has recently been reported and is currently being evaluated in clinical trials [14]. Furthermore, many additional inhibitors are currently being evaluated for their clinical efficacy, including a pan-KRAS inhibitor that targets many of the mutants present in human cancers [15]. Despite this burgeoning success in targeting KRAS, there remain no available inhibitors that target NRAS alone.

As an alternative to development of small molecule RAS inhibitors, we have utilized monobody (Mb) technology to study RAS function and identify novel therapeutic vulnerabilities. We have developed several Mbs that specifically interact with and inhibit specific RAS isoforms and oncogenic mutants [16–18]. For instance, the NS1 Mb binds the α4-α5 allosteric lobes of HRAS and KRAS, but not NRAS, and inhibits RAS function by disrupting RAS nanoclustering, becoming the first experimental reagent capable of perturbing this process [19–21]. Additional RAS inhibitory monobodies have provided unique insights into therapeutic strategies for inhibiting KRAS [11, 22, 23]. These studies exemplify the power of Mb technology to inhibit RAS and to gain new insights into RAS biology.

Selective targeting of NRAS presents challenges in molecular recognition. The effector-binding region of NRAS is identical to those of HRAS and KRAS. At the same time, this region harbors the most common oncogenic mutations of NRAS at positions, 12, 13, and 61. Thus, an ideal inhibitor targeting the effector-binding region would bind multiple NRAS mutants and discriminate NRAS from HRAS and KRAS. While KRAS-specific inhibitors have been described that achieve such selectivity [15], no such NRAS-specific inhibitors have yet been reported. The isoform specificity of the NS1 Mb suggested the feasibility of selective inhibition of NRAS in a mutation-agnostic manner. NS1 binds HRAS and KRAS but not NRAS with the main determinant of this specificity being amino acid 135 in the α4 region, which is Arg in HRAS and KRAS and Lys in NRAS [19]. Thus, we envisioned the possibility of developing new monobodies with a specificity profile inverse of NS1, binding only to NRAS, but not HRAS or KRAS, in a mutation-agnostic manner.

Here, we report the development of a pan NRAS inhibitory Mb, termed Mb(NRAS_24), abbreviated as Mb24 hereafter for brevity. Mb24 inhibited both EGF-stimulated NRAS signaling and oncogenic NRAS signaling and transformation. When tagged with a truncated version of the VHL E3 ligase subunit [22, 24, 25], Mb24 induced NRAS degradation. This study highlights the feasibility of selectively targeting NRAS as a potential therapeutic avenue for NRAS-mutant cancers.

## Results

### Selective binding and colocalization of Mb24 with NRAS

**Fig. 1:**
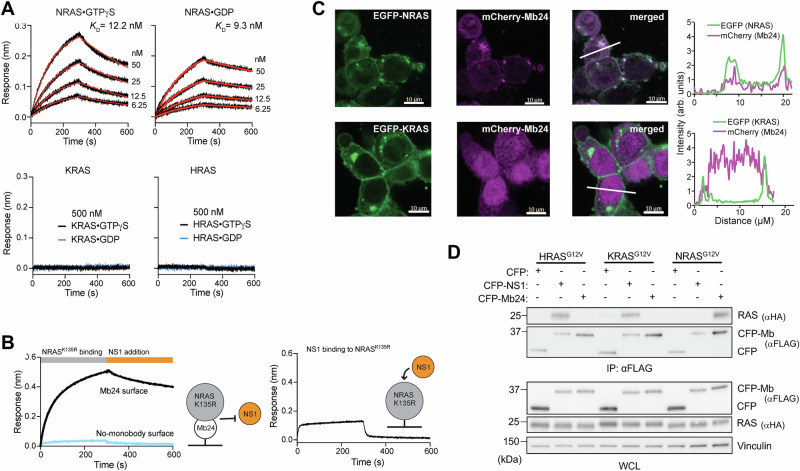
Mb24 specifically interacts with NRAS. **A** Biolayer interferometry (BLI) sensorgrams of RAS isotypes loaded with either GTPγS or GDP to Mb24 immobilized on a sensor tip. The *K*_D_ values shown are from global fitting of a 1:1 binding model. N.D. not determined due to weak binding. **B** Mb24 and NS1 share an overlapping epitope. BLI sensorgram of immobilized Mb24 binding to NRAS(K135R)•GDP binding followed by the addition of NS1 (left graph). NS1 did not bind to the NRAS(K135R) precomplexed with Mb24. NS1 does bind to immobilized NRAS(K135R) in the absence of Mb24 (right graph). **C** Colocalization of mCherry-tagged Mb24 with EGFP-tagged KRAS4B or NRAS in cotransfected HEK 293 cells. The graphs show the fluorescence intensity profiles across the microscopy images. **D** Coimmunoprecipitation of Mbs from HEK 293 cells cotransfected with FLAG-tagged CFP alone, CPF-NS1, or CFP-Mb24 and HA-tagged HRAS^G12V^, KRAS^G12V^, or NRAS^G12V^. Top panel, αFLAG immunoprecipitates were probed with αFLAG and αHA antibodies. Bottom panels, whole cell lysates (WCL) were probed with the indicated antibodies.

We developed monobodies selective for NRAS by following established methods that combine phage and yeast display technologies [11, 22, 26]. To enrich Mb clones that targeted the α4-α5 region of NRAS, the region equivalent to the epitope of NS1, we incorporated positive selection using NRAS•GDP and NRAS•GTPγS and negative selection using HRAS•GDP, HRAS•GTPγS, KRAS•GDP, and KRAS•GTPγS. This campaign led to the development of Mb24, which selectively bound to wild-type NRAS in both GDP- and GTPγS-bound states, but showed no binding to HRAS or KRAS (Fig. 1A). Mb24 binding to NRAS(K135R), a designed mutant that enabled NS1 to bind to NRAS, prevented NS1 binding demonstrating that Mb24 and NS1 likely share an overlapping epitope on NRAS (Fig. 1B).

Next, we tested the binding specificity of Mb24 in cells. mCherry-tagged Mb24 co-localized with EGFP-NRAS but not EGFP-KRAS4B (Fig. 1C) in HEK293T cells. Further, Mb24 selectively co-precipitated with NRAS^G12V^ (Fig. 1D and Supplementary Fig. 1) and NRAS^Q61L^ (Supplementary Figs. 2 and 3) but not HRAS^G12V^, KRAS^G12V^, and KRAS^G12D^. By contrast, NS1 interacted with HRAS and KRAS but not with NRAS mutants as expected (Fig. 1D and Supplementary Figs. 1–3). These experiments demonstrate that Mb24 specifically interacts with NRAS in a mutation-agnostic manner.

### Mb24 inhibits oncogenic NRAS and EGF-stimulated signaling of wild-type NRAS

We next sought to determine whether Mb24 inhibits NRAS-mediated activation of mitogen-activated protein kinase (MAPK) pathway. We utilized the recently developed RASless HEK 293 model cellular system [27], devoid of all three *RAS* genes to focus on specific RAS isoforms or mutants selectively introduced into cells. In contrast to RASless MEFs [28], RASless HEK 293 cells remain viable and proliferative when devoid of RAS. However, MAPK pathway activation upon growth factor stimulation is lost in RASless HEK 293 cells. Lastly, these cells were derived from the Flp-In HEK 293 cell line (Invitrogen), allowing for stable integration of genes of interest to a genomic FRT site.

**Fig. 2:**
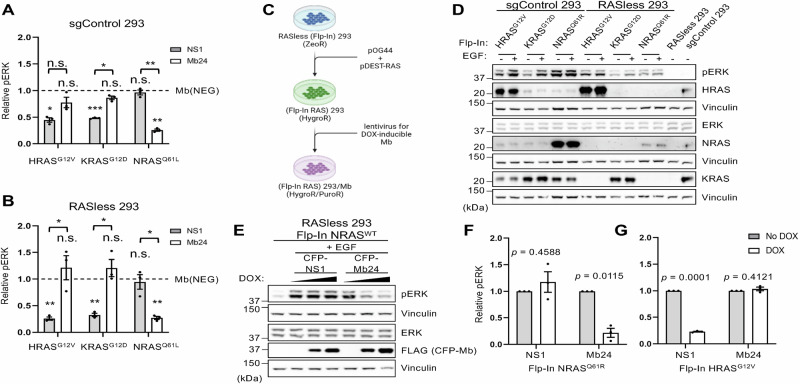
Effect of Mb24 on NRAS signaling in cells. **A** Quantification of pERK/ERK in parental HEK 293 cells as well as (**B**) RASless HEK 293 cells cotransfected with CFP-FLAG-tagged Mb(NEG), NS1 or Mb24 and HRAS^G12V^, KRAS^G12D^, or NRAS^Q61L^. All results were normalized to Mb(NEG). All experiments were repeated at least three times (*n* = 3) and results quantified using Welch’s *t*-test; error bars represent SEM. (***p < 0.0005, **p < 0.005, and *p < 0.05, n.s. not significant). Asterisks under brackets are pERK/ERK values from NS1 or Mb24 compared to Mb(NEG) while those above brackets are comparisons of NS1 versus Mb24. **C** Illustration of the workflow to generate RASless HEK 293 cells which stably express different RAS isoforms/mutants and/or different Mbs. **D** Western blot of control HEK 293 cells (sgControl 293) and RASless HEK 293 cells, which stably express different RAS isoforms/mutants, stimulated with ± EGF (20 ng/mL for 5 min). **E** Effect on pERK of DOX-induced (0, 1, and 10 μg/mL DOX) CFP-FLAG-tagged NS1 or Mb24 in EGF-stimulated RASless HEK 293 (Flp-In wild-type NRAS) cells. **F** Quantification of Mb effect on pERK/ERK in RASless HEK 293 (Flp-In NRAS^Q61R^) and (**G**) RASless HEK 293 (Flp-In HRAS^G12V^) (*n* = 3). Results quantified using Welch’s *t*-test; error bars represent SEM.

Transient expression of HA-tagged HRAS^G12V^, KRAS^G12D^, or NRAS^Q61L^ in RASless HEK 293 cells results in MAPK pathway activation compared to parental cells (Supplementary Figs. 2 and 3). Similar results are seen with the control HEK 293 cells expressing control guide RNAs (sgControl HEK 293) (Supplementary Figs. 2 and 3). Co-expression of CFP-FLAG Mb24 inhibited ERK-MAPK activation only in NRAS^Q61L^-expressing cells (Fig. 2A, B and Supplementary Figs. 2 and 3) whereas NS1 inhibited ERK-MAPK activation only in cells expressing either HRAS^G12V^ or KRAS^G12D^. Mb(NEG), a negative control Mb that does not bind any RAS isoforms, did not inhibit any RAS isoform (Fig. 2A, B and Supplementary Figs. 2–4).

Next, we utilized the Flp-In FRT system to generate cells that stably express single RAS isoforms (Fig. 2C). Western blot analysis of these cells confirmed expression of the indicated RAS isoforms/mutants and restoration of MAPK pathway activity (Fig. 2D and Supplementary Figs. 5 and 6). Next, we generated stable, doxycycline (DOX)-inducible Mb expressing variants of these cells (Fig. 2C). DOX-induced Mb24 expression inhibited EGF-stimulated wild-type NRAS (Fig. 2E and Supplementary Fig. 7) as well as oncogenic NRAS signaling but had no effect on oncogenic HRAS (Fig. 2F, G and Supplementary Fig. 8). Overall, these data demonstrate that Mb24 specifically inhibits signaling mediated by both wild type and oncogenic NRAS.

### Chemical induction of Mb24 in NRAS-mutant tumor lines inhibits signaling and biology

**Fig. 3:**
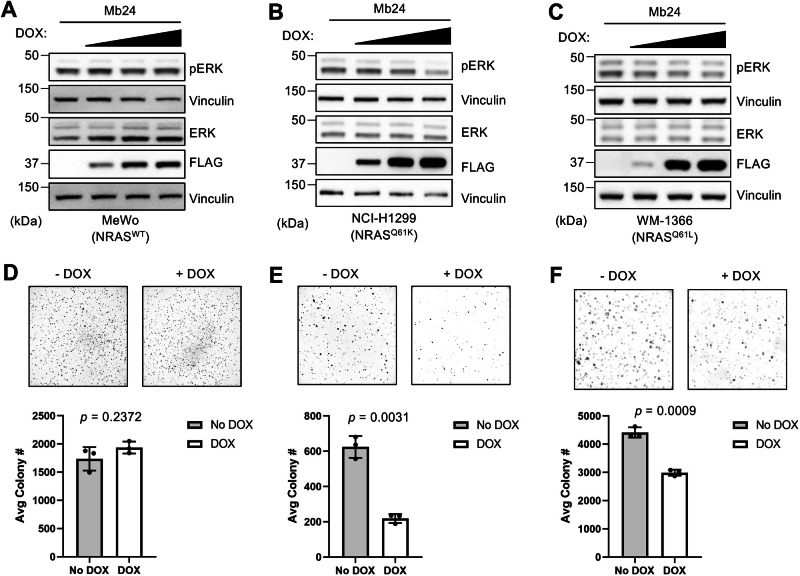
Mb24 activity in tumor cell lines. DOX titration (0, 0.1, 1.0, 10.0 μg/mL) of stable Mb24-expressing tumor cell lines [**A** MeWo (wild-type NRAS), **B** NCI-H1299 (NRAS^Q61K^), **C** WM-1366 (NRAS^Q61L^)]. ERK phosphorylation was quantified using densitometry measurements of pERK/ERK from Western blots (Supplementary Figs. 9 and 10) (*n* = 3). **D**–**F** Soft agar assays to analyze the effect of DOX-induced Mb24 expression on anchorage-independent growth of indicated tumor cell lines (*n* = 3). Colonies from −DOX and +DOX samples were quantified using ImageJ and Welch’s *t*-test was used to compare −DOX and +DOX samples; error bars represent SD.

Next, we assessed the ability of Mb24 to inhibit NRAS-mutant human tumor cells. We generated two melanoma cell lines, MeWo (NRAS^WT^) and WM-1366 (NRAS^Q61L^), and one lung adenocarcinoma line, NCI-H1299 (NRAS^Q61K^), that stably express CFP-FLAG-tagged Mb24 upon DOX treatment. Chemical induction of Mb24 expression in MeWo yielded no detectable decrease in ERK phosphorylation (Fig. 3A and Supplementary Figs. 9A and 10A). In contrast, Mb24 expression decreased ERK-MAPK phosphorylation in NCI-H1299 (Fig. 3B and Supplementary Figs. 9B and 10B) and WM-1366 (Fig. 3C and Supplementary Figs. 9C and 10C). A similar trend was observed in NRAS-mutant neuroblastoma cells, SH-EP, upon induction of Mb24 expression (Supplementary Fig. 11).

To further explore the ability of Mb24 to inhibit oncogenic NRAS function, we tested the effect of Mb24 on the anchorage-independent growth and migration of NRAS-mutant tumor cells. In agreement with the effects on Mb24 on ERK-MAPK activation, DOX-induced Mb24 expression inhibited anchorage-independent growth of NRAS-mutant cell lines but did not impact the growth of wild-type NRAS melanoma cells (Fig. 3D–F). Further, Mb24 reduced the migration of NCI-H1299 cells (Supplementary Fig. 12). These data demonstrate that Mb24 inhibits the biological activity of oncogenic NRAS mutants.

### Mechanism of Mb24-driven NRAS inhibition

**Fig. 4:**
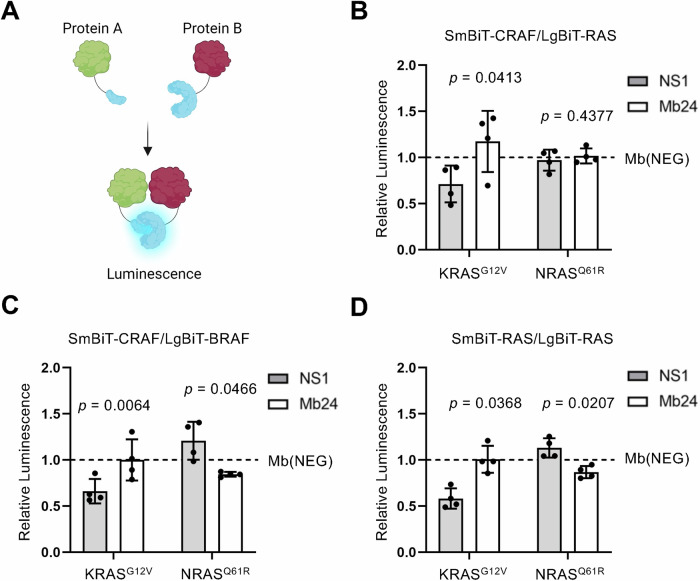
Mechanism of action of NRAS inhibition by Mb24 in live cells. **A** Illustration of the NanoBiT assay used to assess PPIs in live cells. **B** Effect of NS1 (gray) and Mb24 (white) on the interaction of SmBiT-CRAF with LgBiT-KRAS^G12V^ or LgBiT-NRAS^Q61R^. **C** Effect of NS1 and Mb24 on SmBiT-CRAF and LgBiT-BRAF interaction in cells cotransfected with HA-tagged KRAS^G12V^ or NRAS^Q61R^. **D** Effect of NS1 or Mb24 on LgBiT- and SmBiT-tagged KRAS^G12V^ or NRAS^Q61R^ to assess the effect of these Mbs on RAS self-association. All experiments were repeated four times (*n* = 4), normalized to a control Mb (Mb(NEG), represented by the dotted line), and analyzed using a paired *t*-test to compare the effects of NS1 versus Mb24; error bars represent SD.

We next set out to determine the mechanism of action of Mb24-driven NRAS inhibition. Mb24 binds to both GDP- and GTP-loaded NRAS (Fig. 1A) and likely binds in the α4-α5 region of NRAS (Fig. 1B). We therefore hypothesized that Mb24, like NS1, may inhibit NRAS by disrupting NRAS nanoclustering and/or impeding downstream RAF dimerization and activation. One distinction between the effect of NS1 on HRAS versus KRAS is that NS1 disrupts KRAS:RAF interaction but not HRAS:RAF association, a distinction attributed to differences in the hypervariable regions (HVRs) of these RAS isoforms [21]. We therefore analyzed the effect of Mb24 on key protein:protein interactions (PPIs) in the RAS/MAPK signaling pathway, namely RAS:RAF, CRAF:BRAF, and RAS:RAS interactions in live cells using NanoBiT technology (Fig. 4A) [29].

We first tested the effect of Mb24 on KRAS^G12V^ or NRAS^Q61R^ interaction with CRAF. RASless HEK 293 cells were used to eliminate competition with endogenous RAS [29]. NS1 decreased the interaction of LgBiT-KRAS^G12V^ with SmBiT-CRAF compared to Mb24 (Fig. 4B) in agreement with previous reports for NS1 [21]. In contrast, Mb24 had no effect on NRAS^Q61R^ interaction with CRAF (Fig. 4B). These data suggest Mb24 does not affect NRAS interaction with CRAF.

Next, we tested the effect of Mb24 on CRAF:BRAF association, which was shown to be elevated in melanogenic NRAS mutants [30]. As expected, NS1, but not Mb24, decreased CRAF:BRAF association when co-expressed with HA-tagged KRAS^G12V^ (Fig. 4C). In contrast, Mb24, but not NS1, decreased CRAF:BRAF interaction when co-expressed with HA-tagged NRAS^Q61R^, albeit to a lesser extent than seen with NS1 and KRAS (Fig. 4C). These results suggest that Mb24, at least in part, inhibits NRAS-mediated signaling through disrupting oncogenic NRAS-induced CRAF:BRAF heterodimerization.

Lastly, we tested the effect of Mb24 on RAS:RAS association. As shown with reagents like NS1, sterically hindering RAS:RAS association decreases RAF dimerization and activation [19–21, 31]. As expected, NS1 resulted in approximately a 50% reduction in KRAS^G12V^ self-association without affecting NRAS^Q61R^ self-association (Fig. 4D). In contrast, Mb24 did not affect KRAS^G12V^ self-association but did result in a modest, but significant, decrease in NRAS^Q61R^ self-association (Fig. 4D). These results are in agreement with the effect of Mb24 on CRAF:BRAF interactions and imply that Mb24, like NS1, inhibits NRAS through sterically interfering with nanoclustering at the plasma membrane, thereby inhibiting NRAS-stimulated RAF dimerization and activation.

### Inducible NRAS degradation by VHL-tagged Mb24

**Fig. 5:**
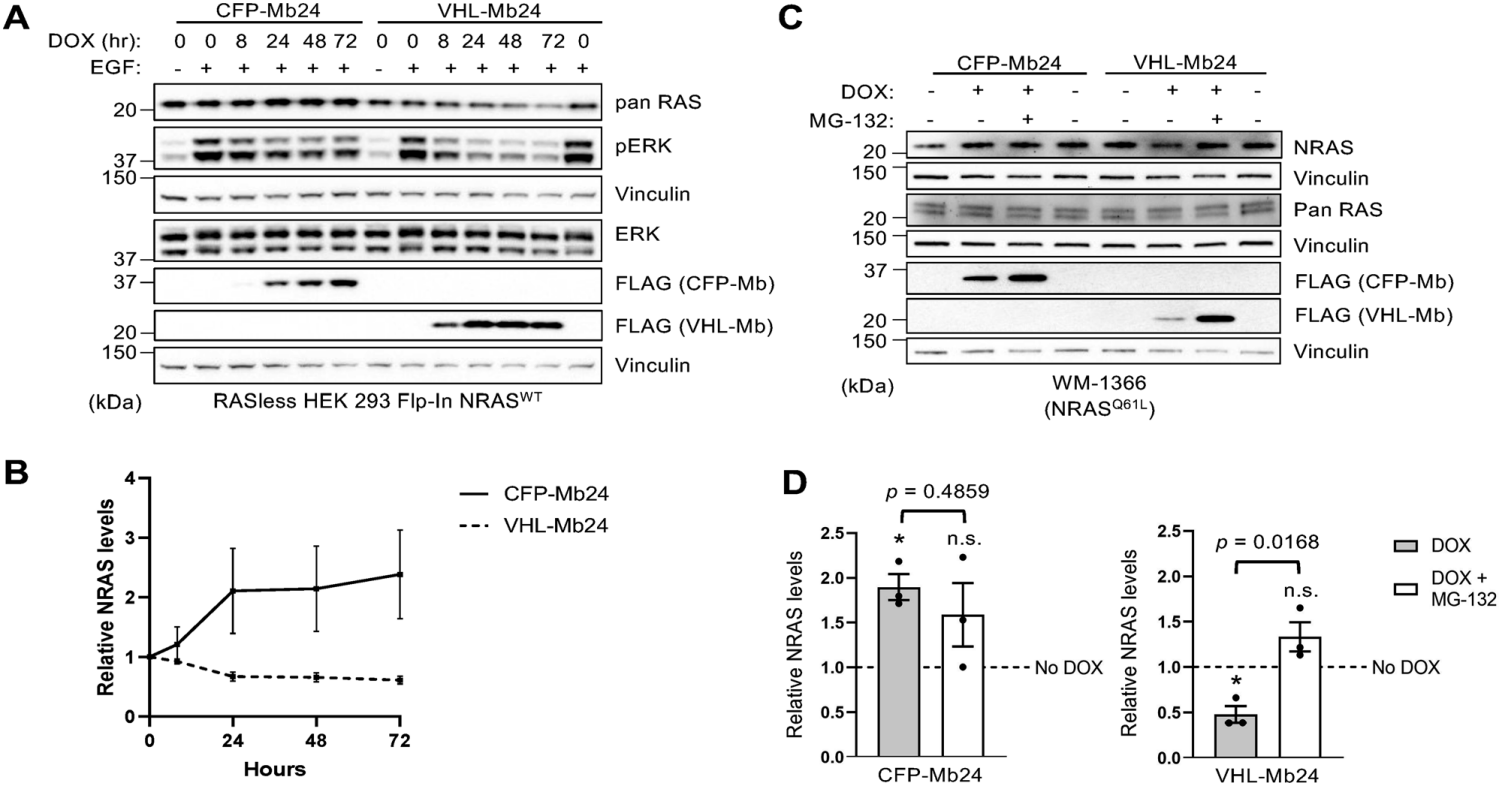
Inducible degradation of NRAS by VHL-Mb24. **A** Expression of CFP-Mb24 or VHL-Mb24 were chemically induced in RASless HEK 293 (Flp-In NRAS^WT^) cells for the indicated time points and then cells were treated with EGF (20 ng/ml) for 5 min before lysate preparation. **B** Quantification of NRAS levels from (**A**). NRAS levels were normalized to vinculin loading control, and all time points were normalized to 0 h of DOX treatment. NRAS levels from each time point were compared to 0-h time points to determine statistical significance using Welch’s *t-*test. Only the 48- and 72-h timepoints for VHL-Mb24 with DOX were statistically different (*p* = 0.046 and 0.025, respectively). Experiments were repeated three times (*n* = 3); error bars represent SEM. **C** Proteasome inhibition with MG-132 rescues NRAS protein levels in WM-1366 cells which express CFP-Mb24 or VHL-Mb24 upon DOX induction. **D** NRAS levels from (**C**) were normalized to vinculin and all values for either CFP-Mb24 or VHL-Mb24 were normalized to No DOX (dotted line). Results quantified were using Welch’s *t*-test; error bars represent SEM. (*p < 0.05, n.s. not significant). Asterisks under brackets represent a significant difference for normalized NRAS values from DOX or DOX + MG-132 compared to No DOX while values above brackets represent comparison of DOX versus DOX + MG-132. Experiments were repeated three times (*n* = 3).

To increase the utility of Mb24 in the study of NRAS biology, we utilized Mb24 as a warhead for NRAS-targeted proteasomal degradation [22, 24, 32]. Briefly, Mb24 was genetically fused with a truncated version of the VHL E3 ligase lacking its natural substrate binding domain (VHL-Mb24) [22]. In RASless HEK 293 cells with stable expression of wild-type NRAS (Flp-In NRAS^WT^), DOX-induced expression of CFP-Mb24 resulted in a slight increase in NRAS levels over time (Fig. 5A, B). In contrast, VHL-Mb24 resulted in a significant decrease in NRAS levels at 48 and 72 h of DOX treatment compared to no DOX (*p* = 0.046 and 0.025, respectively) (Fig. 5A, B and Supplementary Figs. 13 and 14). Furthermore, DOX induction of VHL-Mb24 in WM-1366 decreased endogenous NRAS levels which was rescued by proteasome inhibition (Fig. 5C, D and Supplementary Fig. 15). Examination of ERK phosphorylation levels revealed that VHL-Mb24 lead to a greater inhibition of ERK phosphorylation levels compared to CFP-Mb24 in the Flp-In NRAS^WT^ cells (Fig. 5A). In contrast, we did not observe a significant inhibition of ERK phosphorylation levels in NRAS^Q61L^ mutant WM-1366 cells (Supplementary Fig. 16). These data indicate that VHL-Mb24 induces proteasome-dependent NRAS degradation whereas CFP-Mb24 stabilizes NRAS levels in cells.

## Discussion

Here, we report the development and characterization of the first NRAS-specific inhibitory “tool biologic” [33], Mb24, that selectively interferes with NRAS, but not H- or KRAS, activity in a mutation and nucleotide agnostic fashion. Mb24 joins an arsenal of previously developed RAS inhibitory Mbs with distinct specificities (Supplementary Table 1) [11, 19, 22, 23, 34]. Despite the success in pharmacologically inhibiting KRAS, NRAS has remained recalcitrant to inhibition. Mb24 represents the first reported NRAS-selective inhibitor, targeting both wild-type and oncogenic forms of NRAS in both nucleotide states to reduce signaling and impair the biologic activity of oncogenic NRAS (Figs. 1, 2). Although the effect of Mb24 on oncogenic NRAS-mediated activation of ERK in melanoma cells is rather modest, these findings are in agreement with prior published studies [35].

The mechanism by which Mb24 inhibits NRAS appears to mirror the activity of NS1 in binding the allosteric lobe to disrupt HRAS and KRAS nanoclustering (Fig. 1B) [18]. Indeed, Mb24 reduces NRAS self-association albeit to a lesser extent than was seen with NS1 on KRAS self-association (Fig. 4D). Our previous work demonstrated that mutations in the α4-α5 region of NRAS were not sufficient to inhibit NRAS signaling activity [29]. Our current work further supports the notion that nontraditional therapeutic approaches, such as macromolecules (i.e., monobodies) or molecular glues, would be necessary to inhibit RAS isoforms when targeting RAS multimerization.

The lack of effect of Mb24 on NRAS:RAF interaction also mirrors the non-effect of NS1 on HRAS:RAF interaction. This may be due to the similarity of the HRAS and NRAS HVRs. While all RAS isoforms undergo obligate prenylation at the C-terminal CAAX site, a secondary signal in the HVR is needed to target RAS isoforms to distinct membrane domains. For HRAS, two additional Cys residues are palmitoylated to serve this purpose while KRAS utilizes a poly-basic region. The NRAS HVR more closely resembles that of HRAS but with only a single Cys as a palmitoylation site. These secondary palmitoyl modifications of the HVRs of NRAS and HRAS may orient the GTPase domains differently from that of KRAS resulting in the different effects of NS1 on RAF interaction with KRAS versus HRAS and NRAS [21].

We also developed an NRAS-specific biomolecular degrader by linking Mb24 to a truncated version of the VHL E3 ligase that lacks its natural substrate binding domain as this was previously shown to be an effective method for other biologics [22, 24, 25, 36]. We hypothesized that compared to CFP-Mb24, targeted degradation of NRAS would lead to a more sustained inhibition of ERK-MAPK activation by NRAS as we previously observed with a VHL-12V/C Mb fusion [22]. CFP-Mb24 lead to an increase in NRAS protein level suggesting a potential stabilizing effect on NRAS. In contrast, VHL-Mb24 decreased NRAS levels which were rescued by proteasome inhibition, confirming the efficacy of this biodegrader at targeting NRAS. Although we anticipated that VHL-Mb24 would result in a more sustainable inhibition of ERK-MAPK pathway activity compared to CFP-Mb24, our results were mixed. VHL-Mb24 reduced ERK phosphorylation levels following EGF stimulation of NRAS expressing RASless HEK cells (Fig. 5A and Supplementary Fig. 14) but did not affect ERK phosphorylation levels in NRAS^Q61L^-mutant WM-1366 melanoma cells (Supplementary Fig. 16). This was a surprising observation as genetic ablation of mutant NRAS in vivo decreased phosphorylated ERK levels and led to tumor regression [37]. Nonetheless, many of these tumors recurred and showed increased RTK signaling as a resistance mechanism [37]. Additionally, previous work by Bond et al. suggest that the effects between targeted degraders and inhibitors on cell viability and signaling may be cell-line dependent [38]. Further work will be necessary to elucidate the differences between inhibition and degradation of NRAS.

In conclusion, we report here the first NRAS-specific binding reagent capable of inhibiting and degrading NRAS in a mutation-agnostic manner. This work indicates that similar to HRAS and KRAS, NRAS is amenable to inhibition through Mb binding to a region outside the effector-binding region. Mb24 joins a group of RAS inhibitory Mbs that have been used in recent years to gain valuable insights into RAS biology and therapeutic vulnerabilities in vitro and in vivo (Supplementary Table 1). Thus, Mb24 will be useful for further addressing RAS biology for translation into novel therapeutic approaches against oncogenic NRAS mutants.

## Materials and methods

### Development of Mb(NRAS_24)

The expression vectors for RAS were described previously using the pHBT vector in such a way that His6 and Avi-tags were attached N-terminal to residues 1–174 of RAS [34]. They were purified using NTA affinity resin (Cytiva) followed with gel filtration using Superdex S75 (Cytiva).

Mb24 was developed using established methods described previously [19, 34]. Briefly, after four rounds of phage-display library selection, yeast display libraries were constructed using the enriched phage-display pool. After three rounds of library sorting in the yeast display format using 200 nM, 200 nM, 100 nM NRAS for the 1st, 2nd, and 3rd rounds, respectively, individual clones were examined in the yeast display format for binding to NRAS, HRAS, and KRAS4B. The expression vector for Mb24 was constructed with the N-terminal tags, and the protein was prepared in the same manner as the RAS proteins.

### Cell culture and cloning

Cells used in each experiment were freshly thawed or generated and routinely tested for mycoplasma. HEK 293 cells (MUSC Tissue Culture Facility) were maintained in Dulbecco’s modified Eagle’s medium (DMEM) (Corning) supplemented with 10% fetal bovine serum (FBS). RASless HEK 293 and sgControl HEK 293 cells were maintained in DMEM supplemented with 10% FBS and 100 μg/mL Zeocin. Flp-In RAS HEK 293 lines were maintained in DMEM supplemented with 10% FBS and 200 μg/mL of Hygromycin B. All HEK 293 cell lines transduced with DOX-inducible Mbs were maintained in the cell line’s normal media with tetracycline-free FBS plus 0.5–1.0 μg/mL puromycin. The melanoma cell line MeWo was maintained in DMEM supplemented with 10% FBS. WM-1366 and WM-1361A melanoma cell lines were maintained in RPMI media supplemented with 5% FBS. Lastly, the lung adenocarcinoma line NCI-H1299 was maintained in RPMI media supplemented with 10% FBS. All cancer cell lines were maintained in regular base media with tetracycline-free FBS and 1 μg/mL puromycin when transduced with DOX-inducible Mbs. Tumor cell lines were intermittently authenticated by STR profiling.

All HA-tagged RAS and CPF-FLAG-tagged Mb expression vectors were constructed as previously described [17]. Gateway cloning vectors (pEF5-FRT-V5-DEST and pENTR-RAS constructs) as well as the Flp-Recombinase expression vector pOG44 were kindly provided by the lab of Frank McCormick. Expression clones encoding different RAS isoforms and mutants (pDEST-RAS) were made using Gateway LR Clonase II (ThermoFisher #11791020) with indicated pENTR-RAS constructs and pEF5-FRT-V5-DEST per manufacturer protocol. All NanoBiT RAS expression vectors (SmBiT/LgBiT-tagged RAS) were kindly donated from Matt Robers and Jim Vasta (Promega©). The SmBiT-CRAF-LgBiT-BRAF dual expression vector was kindly donated by Christin Burd. The SmBiT-CRAF expression vector was constructed by PCR amplifying SmBiT-CRAF from the SmBiT-CRAF-LgBiT-BRAF dual expression vector and cloning the PCR fragment into a CMV-driven expression vector. For this, we restriction digested the CFP-FLAG-NS1 sequence from pECFP-FLAG-NS1 and cloned the PCR fragment for SmBiT-CRAF downstream of the CMV promoter. VHL-Mb24 was generated by PCR amplifying a fragment of VHL and, separately, Mb24, then using these fragments to clone into the same empty vector which was used for SmBiT-CRAF cloning. For DOX-regulated expression, all desired sequences were subcloned into the mammalian expression lentiviral vector pCW57.1 (Addgene # 41393).

### Transfections, cell signaling, and NRAS degradation assays

Transfections and cell signaling assays were essentially done as previously described [17]. We typically use 3 μl of polyethylenimine (PEI) (10 mg/mL dissolved in 30% ethanol) for every 1 μg of DNA transfected. Appropriate amounts of PEI and DNA are incubated for 30 min at room temperature in Opti-MEM Reduced Serum Media (Gibco) that is 1/10 the final volume of the media that will be added to cells (1 ml Opti-MEM for 10 cm plate that will contain 10 ml total). This mixture is then added to appropriate cells with serum-free media making up the rest of the volume. For HEK 293 cells, the DNA incubates (at 37 °C and 5% CO_2_) with the cells for 3 h in serum-free media. This media is then aspirated and replaced with complete media for the indicated time points.

For cell signaling assays, we analyzed RAS-mediated ERK activation. For all HEK 293 cell transfections, lysates were collected 48–72 h post transfection of indicated RAS and Mb constructs. For signaling assays in Mb-transduced tumor cell lines, Mb expression was induced with DOX for 48–72 h (unless otherwise indicated) before collecting lysates. ERK and pERK levels were analyzed via Western Blot using α-ERK (Cell Signaling Technology) and α-pERK (Cell Signaling Technology) antibodies. ERK and pERK protein levels were quantified using Image Studio Lite (Ver 5.2) software. pERK/ERK ratios were determined for each experiment and normalized to controls where indicated.

For NRAS degradation assays, indicated cells were transduced with lentivirus to stably express VHL-Mb24 under DOX regulation. DOX was used to induced Mb expression, as explained above, for indicated time points. NRAS levels were quantified from Western Blots and normalized to vinculin loading control using Image Studio Lite (Ver 5.2) software. For experiments involving proteasome inhibition, MG-132 (2.5–5.0 μM) was added to the media at the same time as DOX and incubated with the cells for 24 h before collecting lysates for sample preparation.

### Immunoblotting and antibodies

Sample preparation and immunoblotting were performed as previously described [17, 29]. The following antibodies were used: monoclonal HA (clone 16B12, Biolegend #90154), monoclonal FLAG (Clone M2, Sigma #F1804), phospho-ERK (Thr202/Tyr204, CST #9101), total ERK (CST #9102), Vinculin (SC #73614), CRAF (BD Biosciences #610151), BRAF (Santa Cruz sc-5284), pan RAS (Santa Cruz sc-32), NRAS (Abcam ab167136), HRAS (ProteinTech #15531-1-AP), KRAS (Millipore Sigma #WH0003845M1).

### Generation of Flp-In RAS HEK 293 cell lines

The RASless and sgControl HEK 293 cells were derived from Invitrogen’s Flp-In HEK 293 cell line (Invitrogen #R75007). These cells have stable integration of an FRT/ZeoR site at a transcriptionally active locus. Flp-In vectors, such as pEF5-FRT-V5-DEST, integrate to FRT sites when co-expressed with pOG44 (Flp-Recombinase) in FRT-containing cells. To Generate Flp-In RAS cells, pDEST-RAS expression vectors were cotransfected in a 1:1 ratio with pOG44 into RASless or sgControl HEK 293 cells. Successful Flp-Recombinase-mediated recombination of the pDEST-RAS expression cassette with the FRT site will disrupt the ZeoR gene; the cells will then become sensitive to Zeocin and resistant to Hygromycin B. For this reason, cells were transfected and maintained in antibiotic-free media until 48 h post transfection. Media was thereafter supplemented with 200 μg/mL of Hygromycin B and cells remained under selection until colonies became visible. All Hygromycin B-resistant cells should be isogenic as there is only a single integration of FRT/ZeoR, therefore colonies for each indicated Flp-In RAS line were pooled and expanded. Expression of indicated RAS isoforms and mutants were validated via Western Blot using isoform-specific antibodies, analyzing signaling in the absence and presence of EGF, and also by the use of inhibitory Mbs specific for different RAS isoforms.

### Soft agar colony formation assays

Soft agar colony formation assays were performed as previously described [11, 17]. A solidified base agar layer (0.5%) was topped with cell suspension in 0.33% soft agar and allowed to set. Cells were treated with DOX 1–2 times per week by drop-wise addition of supplemented media to top layer to induce Mb expression. Two to four weeks after plating (until colonies are visible to eye) cells were stained using MTT (100 ml of 2 mg/ml solution of MTT per well). Colony number and average colony size were quantified using ImageJ. Data represent three replicates per condition.

### Migration assays

Transwell migration assays were performed using a reusable multi-well chemotaxis chamber (Neuro Probe #AP48). H1299^Q61K^ and H1299^Q61K^-Mb24 cells were pre-treated for 24 h with 4 μg/ml of DOX prior to the assay; control groups without treatment were also analyzed. Each well contained 10,000 cells in RPMI with 2% FBS (with and without DOX) in the upper chamber, and in RPMI with 20% FBS in the lower chamber. Cells were allowed to migrate for 24 h, fixed with 4% PFA, stained with crystal violet, photographed, and counted. Each condition was performed in duplicate with six counted fields in total per experiment and three biological replicates.

### Live cell NanoBiT protein–protein interactions assays

For all NanoBiT assays, RASless HEK 293 cells were plated in 96-well, white-wall, clear-bottom tissue culture plates were used (ThermoFisher #165306). Unlike other experiments involving transfections, we transfected these cells overnight (reverse transfection) with the indicated DNA vectors the day that the cells were split and plated in the 96-well plates. Briefly, DNA was prepared using PEI as previously described. While the DNA incubated, 4.0 × 10^4^ cells (in 100 µl of complete media) per well were added to appropriate number of wells of a 96-well plate. Each transfection was done in technical triplicates—this was repeated for a total of four biological replicates. After cells were added to wells and DNA/PEI/Opti-MEM incubated for the appropriate time, serum-free media was added to the tubes of DNA to bring the volume to 300 μl total. Then, 100 µl of the appropriate DNA mix was added to indicated wells in triplicate. The cells were incubated overnight before carefully aspirating the media the next day and replacing it with complete media. Twenty-four hours later (~48 h post transfection), media was aspirated from wells, and luminescence was measured using NanoGlo® Live-Cell Substrate (Promega; Cat # N2012) suspended in Opti-MEM® reduced serum media (Gibco; cat # 31985070). After the live-cell luminescence measurement, cells were lysed with 0.1% Triton X-100 and incubated with HiBiT peptide (0.1 µM) for 10 min on orbital shaker at 4 °C. Then, luminescence was measured to quantify LgBiT peptide levels. Live-cell luminescence was normalized to luminescence after HiBiT peptide addition. Luminescence from wells transfected with pECFP-FLAG-NS1 and Mb24 were normalized to Mb(NEG) and then compared to one another. Assays were performed four times each (*n* = 4).

### Statistical analyses

All statistical analyses were performed using GraphPad Prism 9 software. Experiments with statistical analyses were performed at least three times and variance was represented by standard error of the mean (SEM) or standard deviation (SD) and indicated in figure legends. After the appropriate and indicated normalizations, statistical significance between two groups was determined using paired *t*-test or Welch’s *t*-test. In this study, *p* values < 0.05 were considered statistically significant. Asterisks (*) were used in figures to denote statistical significance unless otherwise noted in figure legends. Non-significant changes were either indicated by n.s. or are otherwise not called out in the figures.

## Supplementary information

Supplemental Figures and Table

## Data Availability

Data are available upon request from the authors.

## Materials availability

Materials are available upon request from the authors.
