# Supplementary material for: Inhibition and degradation of NRAS with a pan-NRAS monobody
Source: Oncogene. 2024 Oct 8;43(48):3489–97. doi: 10.1038/s41388-024-03186-y (PMC11584388; doi:10.1038/s41388-024-03186-y)
Supplement: Supplementary file 1 — Supplemental Figures and Table [file 41388_2024_3186_MOESM1_ESM.pdf]

Supplemental Figure 1

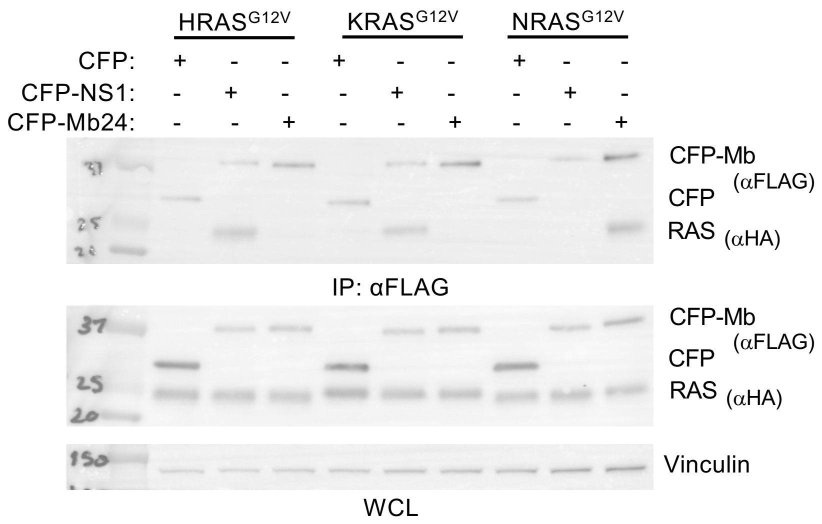

Supplemental Figure 1. Raw data (chemiluminescence overlayed on membrane) from Western Blot from Fig. 1B.

Supplemental Figure 2

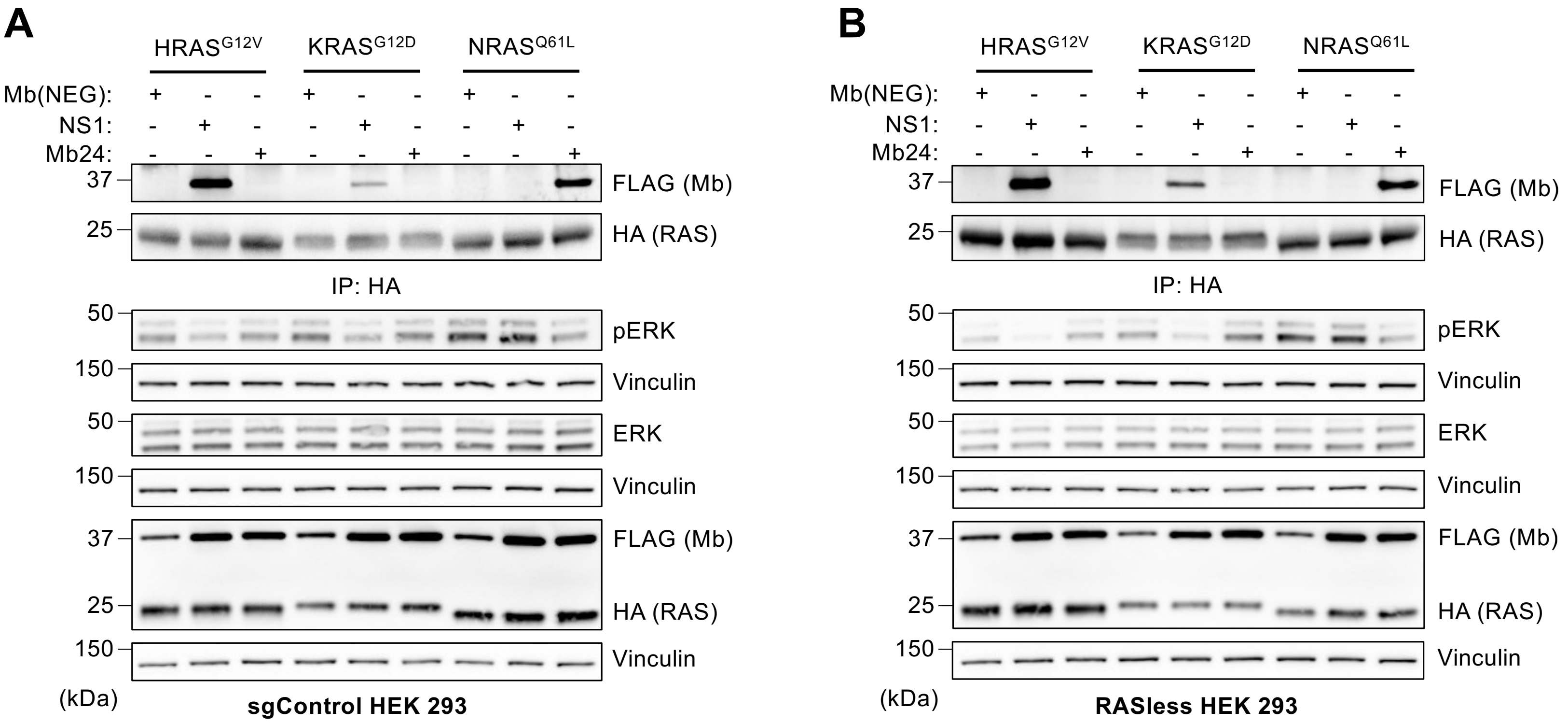

Supplemental Figure 2. Representative Western Blots for Figures 2A and 2B. A) Control (sgControl) HEK 293 cells and (B) RASless HEK 293 cells cotransfected with CFP-FLAG-tagged Mb(-), NS1, or Mb24 and HA-tagged HRAS^{G12V}, KRAS^{G12D}, NRAS^{Q61L}. Western Blots were performed for WCL and immunoprecipitated HA to determine interaction of HA-tagged RAS with indicated FLAG-tagged Mbs.

Supplemental Figure 3

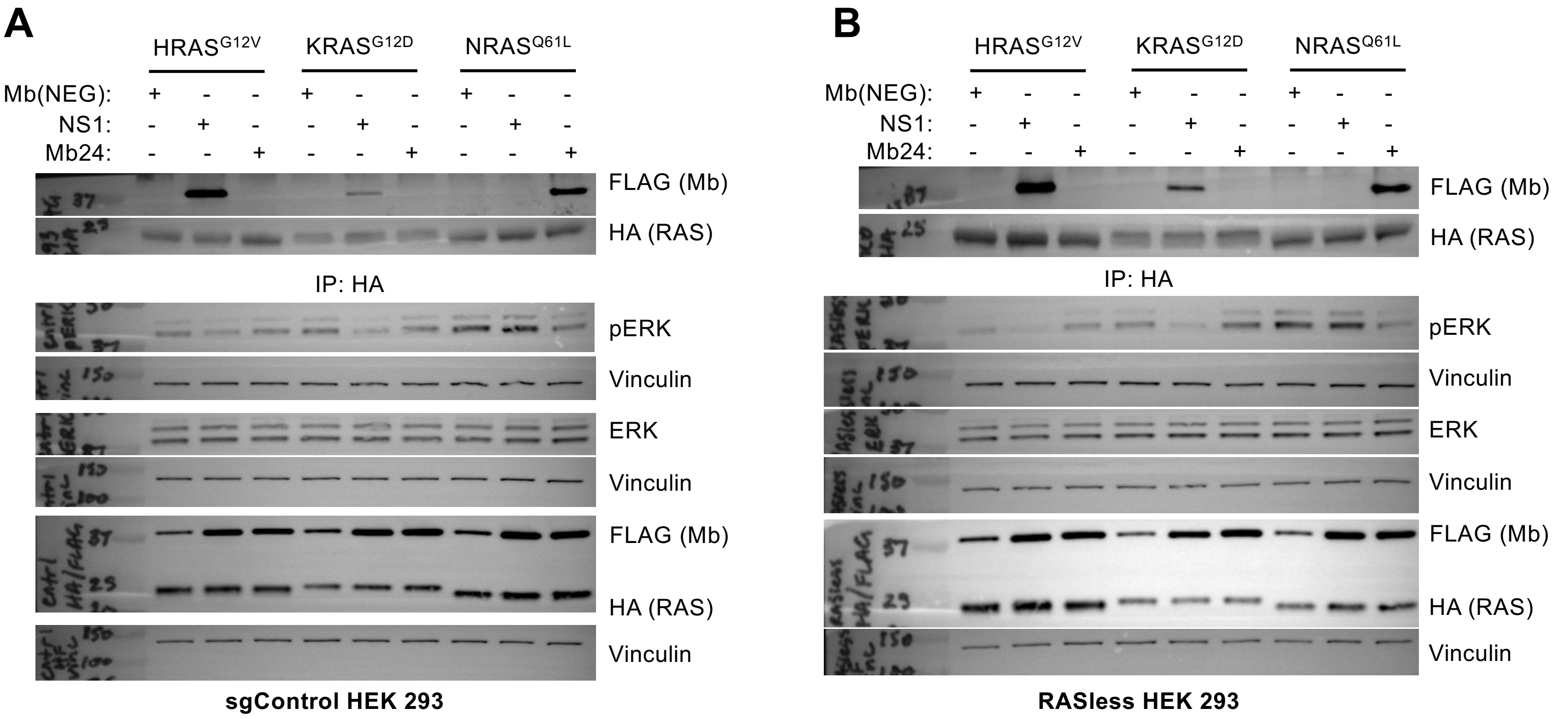

Supplemental Figure 3. Raw data for Sup. Fig. 2.

Supplemental Figure 4

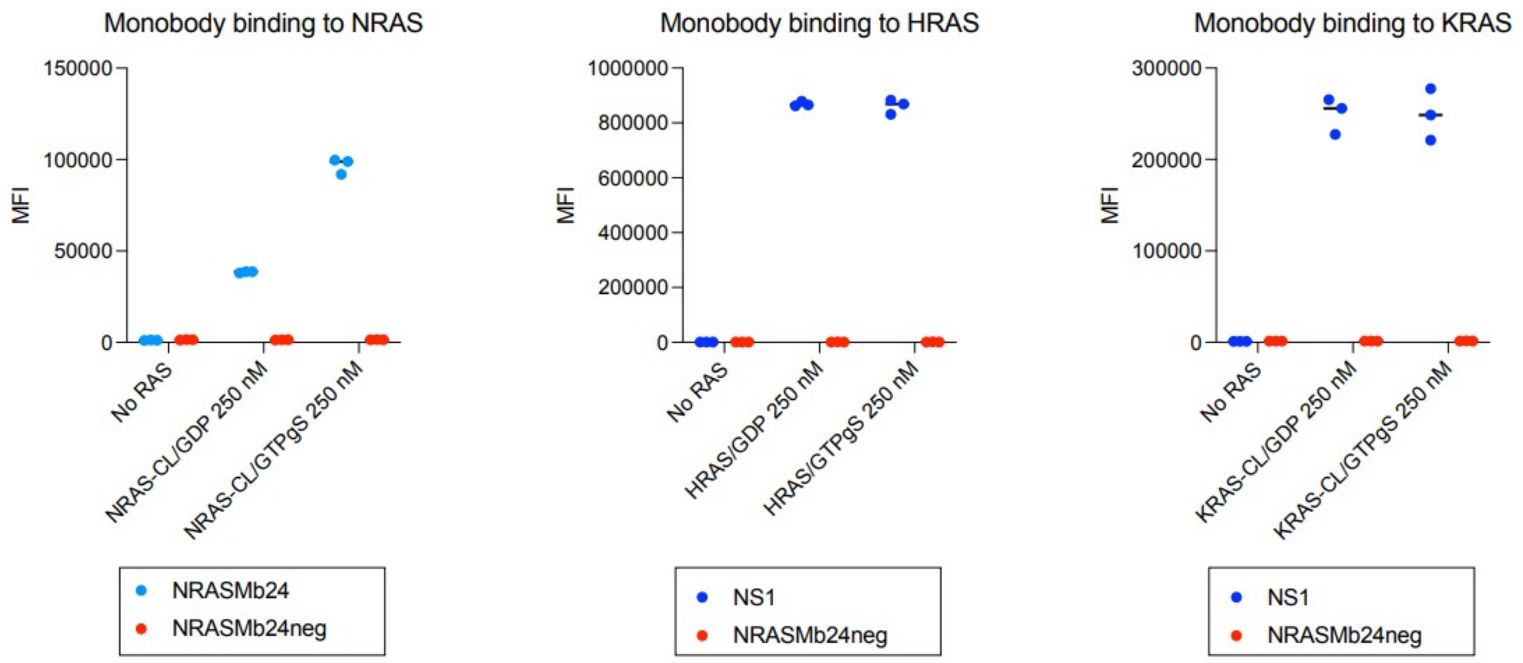

Supplemental Figure 4. Validation of negative control Mb (Mb(-)) that is null for interacting with all RAS isoforms. Results shown are from yeast surface display followed by flow cytometry to determine binding displayed in mean fluorescence units.

Supplemental Figure 5

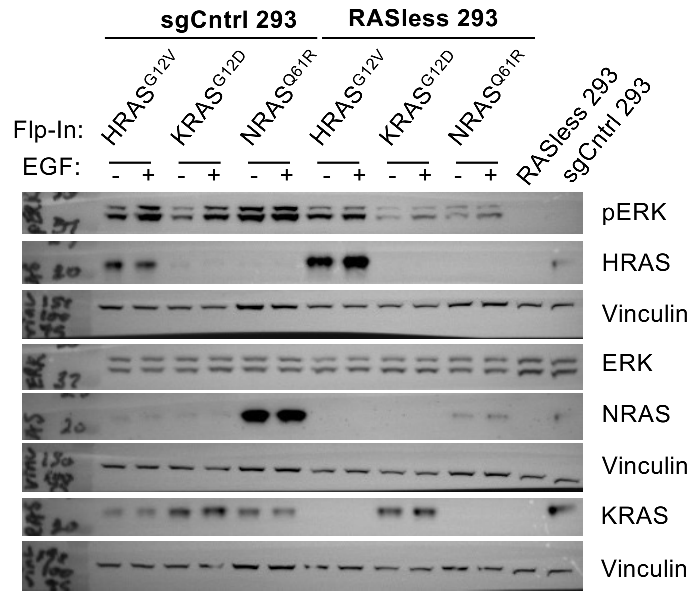

Supplemental Figure 5. Raw data for Figure 2D.

Supplemental Figure 6

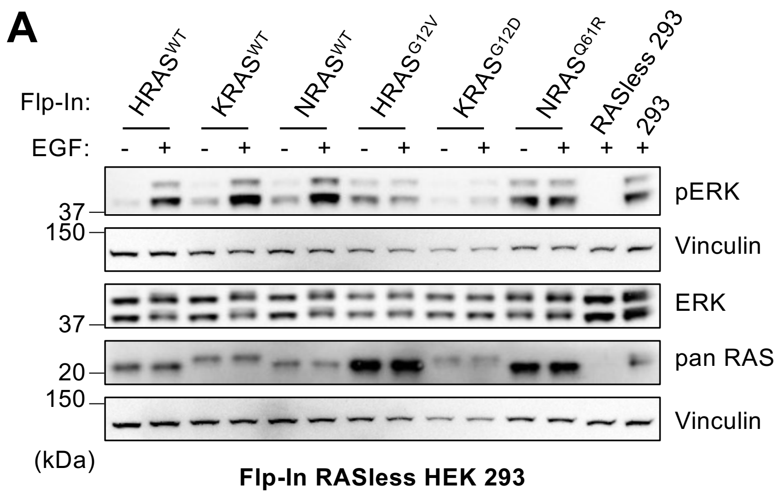

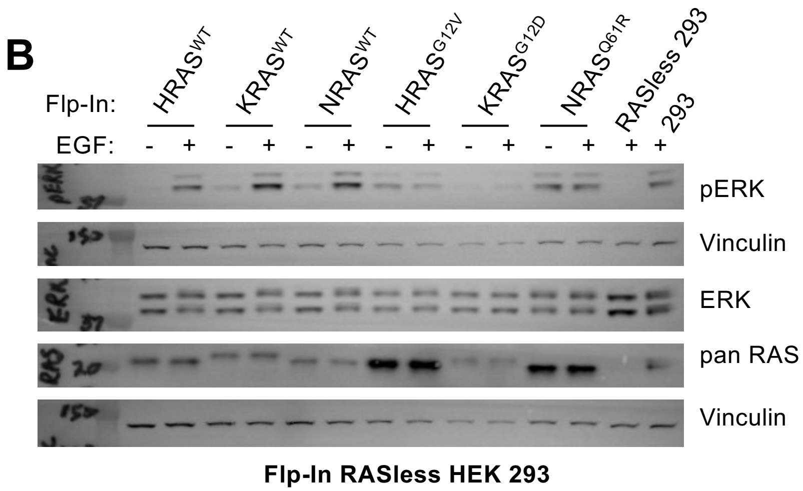

Supplemental Figure 6. A) Western Blot of the indicated RASless HEK 293 (Flp-In RAS) cells -/+ 20 ng/mL EGF for 5 min. B) Raw data for Sup. Fig. 6A.

Supplemental Figure 7

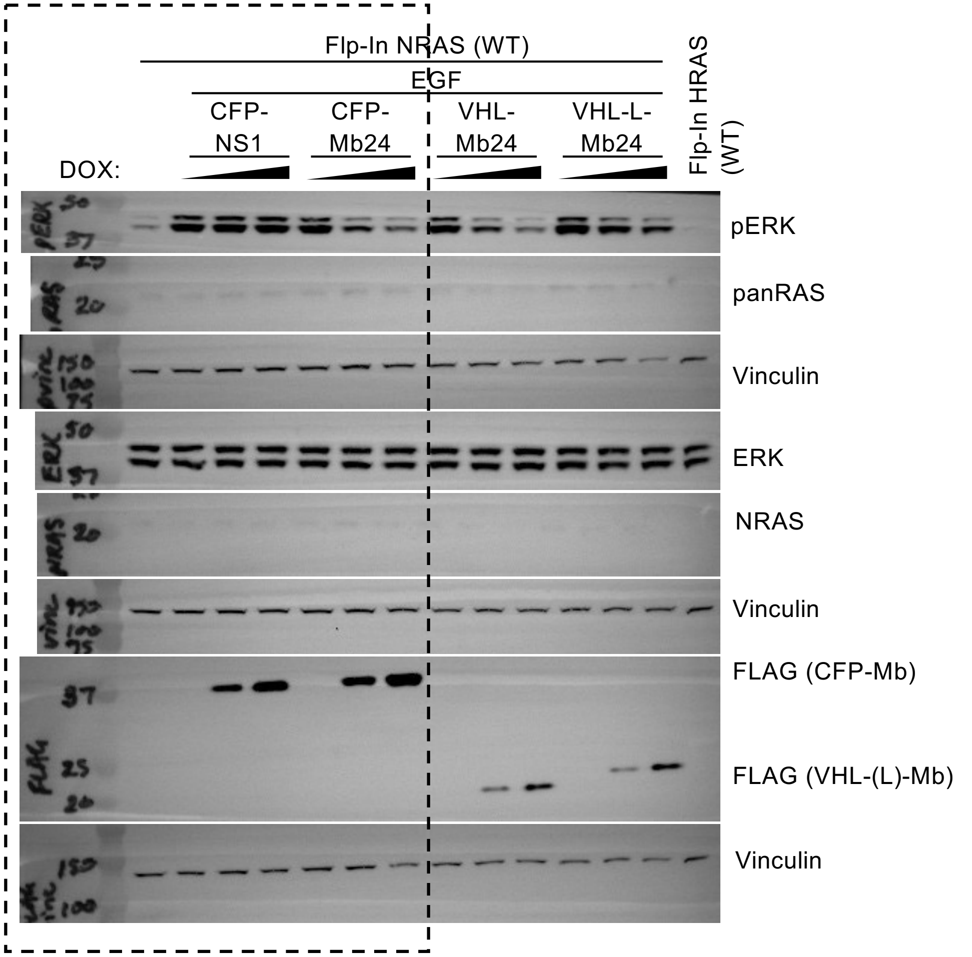

Supplemental Figure 7. Raw data for Fig. 2E and Sup. Fig. 12. Data within the dotted box represents raw data for Fig. 2E while the whole blot represents raw data for Sup. Fig.12.

Supplemental Figure 8

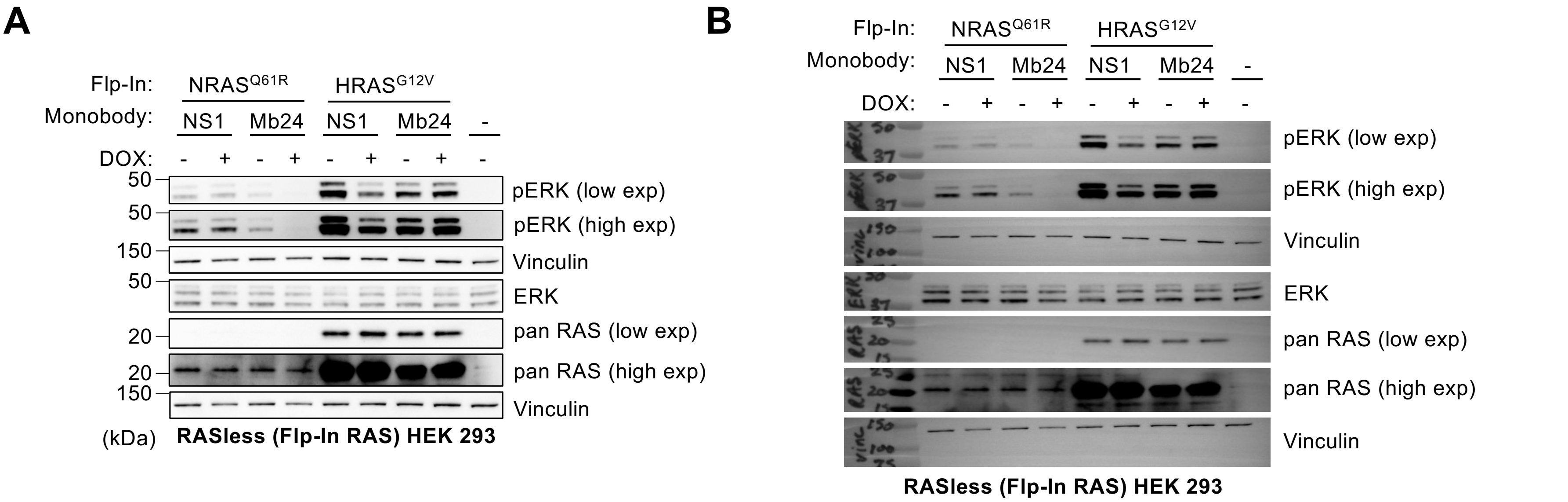

Supplemental Figure 8. A) Western Blot of the indicated RASless HEK 293 (Flp-In RAS) -/+ DOX (2 ug/mL) with stable, DOX-inducible CFP-FLAG-tagged NS1 or Mb24. B) Raw data for Sup. Fig. 8A.

Supplemental Figure 9

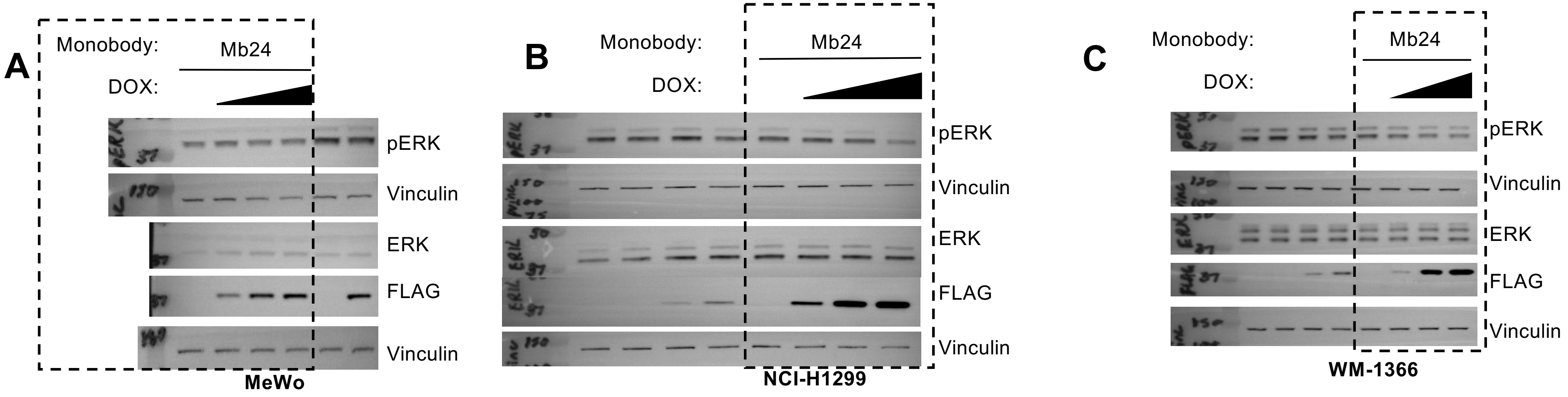

Supplemental Figure 9. Raw data (within the dotted boxes) for Western Blots from Figures 2A–2C.

Supplemental Figure 10

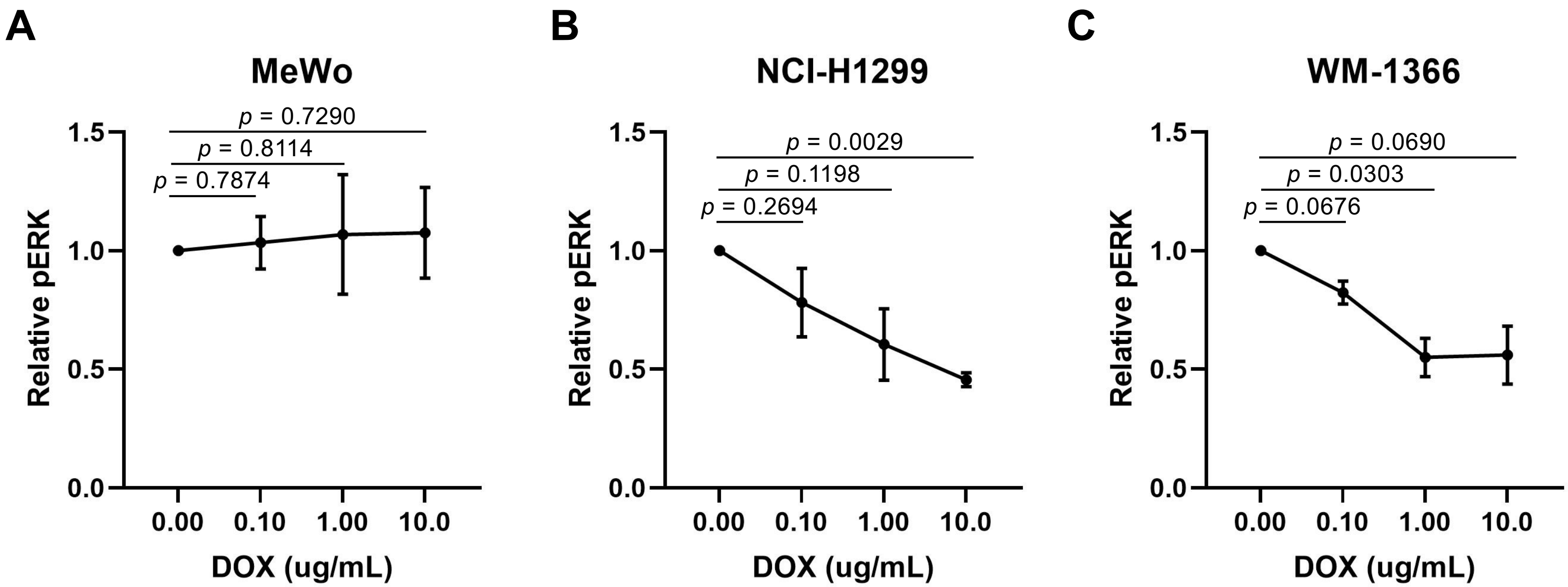

Supplemental Figure 10. Quantification of pERK/ERK from Western Blots of A) Fig. 2A, B) Fig. 2B, and C) Fig. 2C. Results repeated three times each (n=3), normalized to 0.00 ug/ul DOX, and compared using paired t test; error bars represent S.E.M.

Supplemental Figure 11

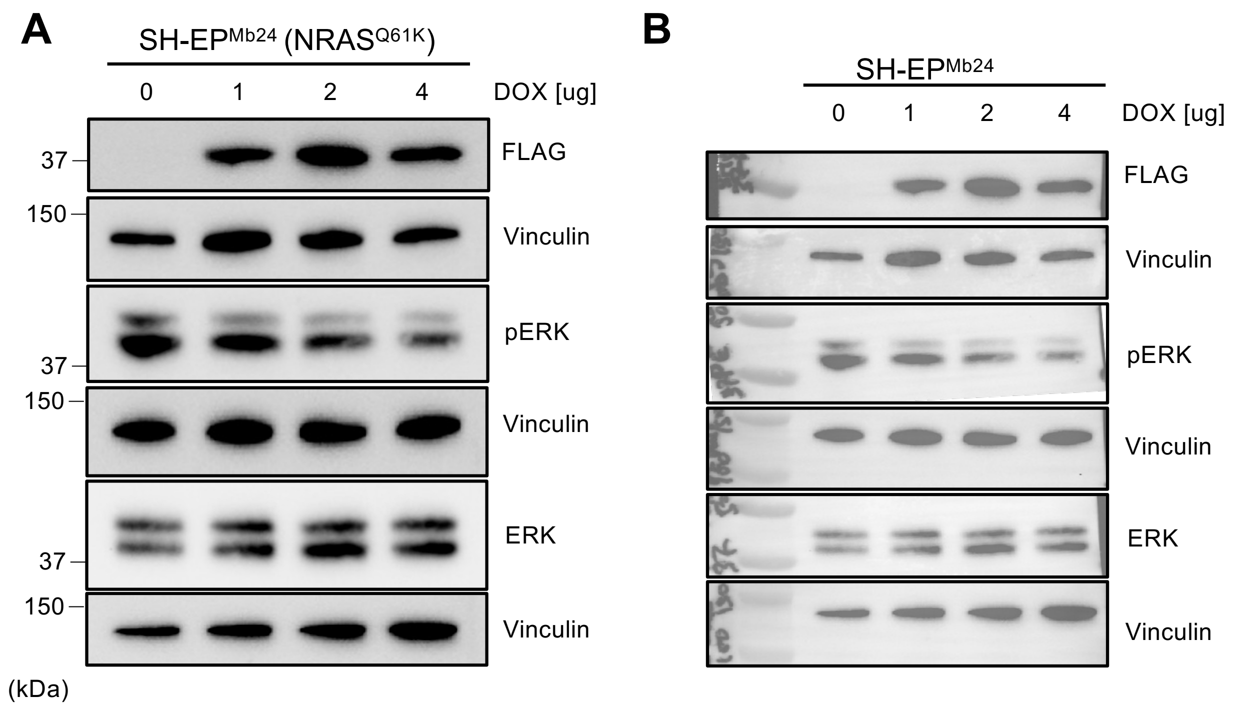

Supplemental Figure 11. Chemically-induced expression of CFP-FLAG-tagged Mb24 in an NRAS-mutant, neuroblastoma cell line, SH-EP, inhibits downstream phosphorylation of ERK. A) Western Blot of SH-EP cells after induced CFP-FLAG-tagged Mb24 expression using the indicated DOX concentrations. B) Raw data for Sup. Fig. 11A.

Supplemental Figure 12

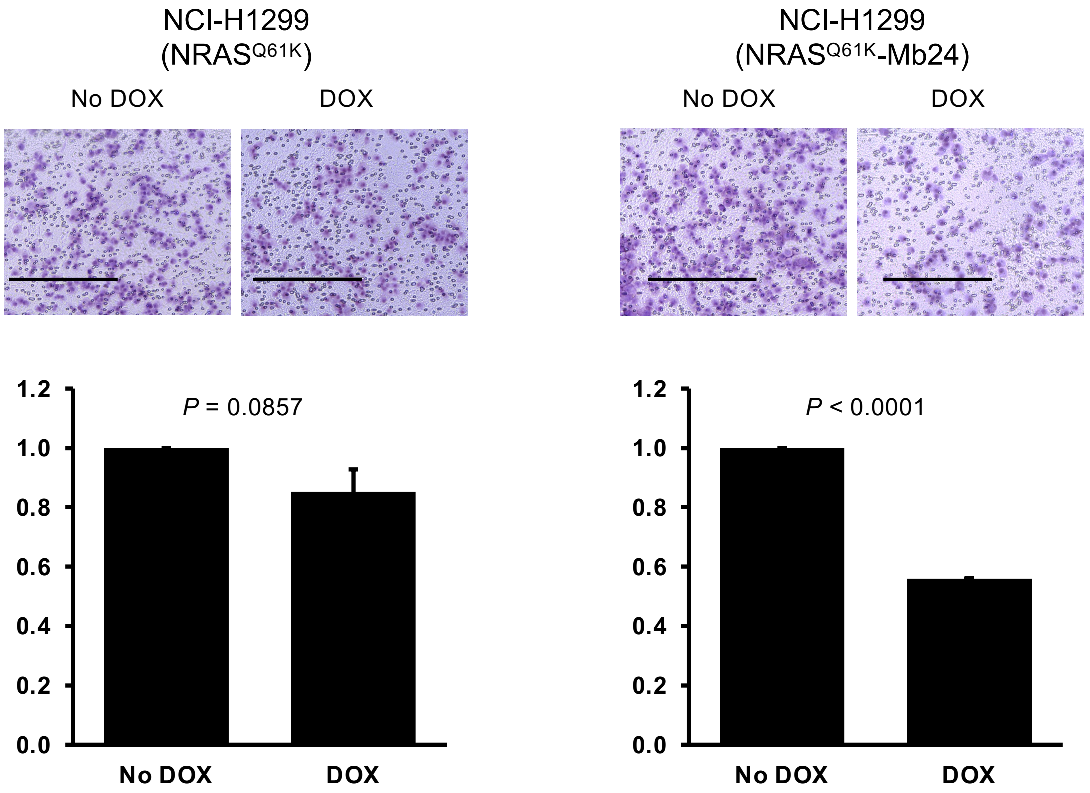

Supplemental Figure 12. Migration assays to assess the effect of DOX-induced Mb24 expression on cell migration. Cell counts from -DOX and +DOX were quantified using ImageJ and t-test (Graph Pad) was used to compare differences between samples. Error bars represent S.E.M.

Supplemental Figure 13

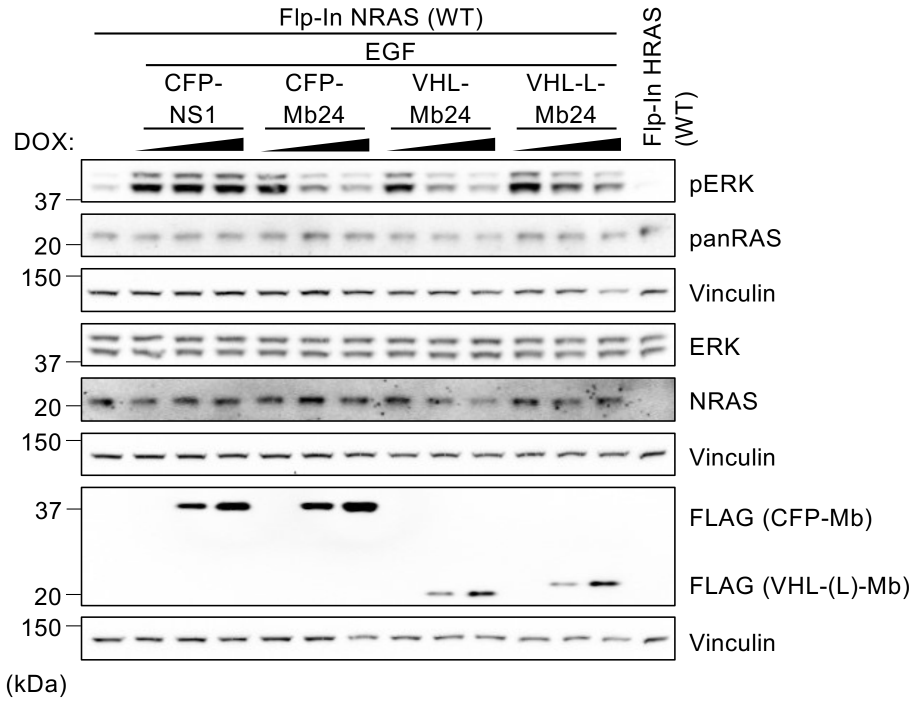

Supplemental Figure 13. Chemically induced VHL-tagged Mb24 inhibits and degrades EGF-stimulated NRAS. Raw data for this figure is shown in Supplementary Fig. 7.

Supplemental Figure 14

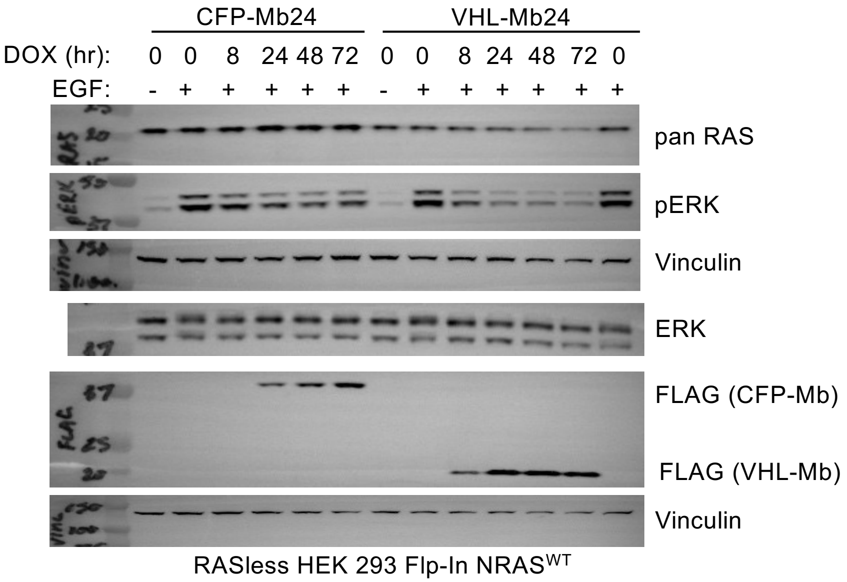

Supplemental Figure 14. Raw data for Western Blot in Fig. 5A.

Supplemental Figure 15

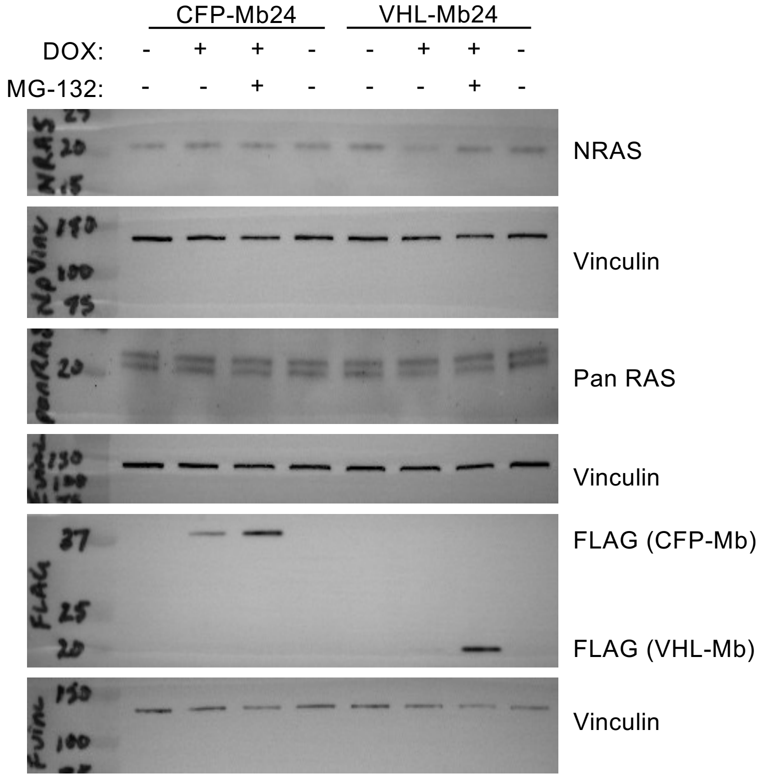

Supplemental Figure 15. Raw data for Western Blot in Fig. 5C.

Supplemental Figure 16

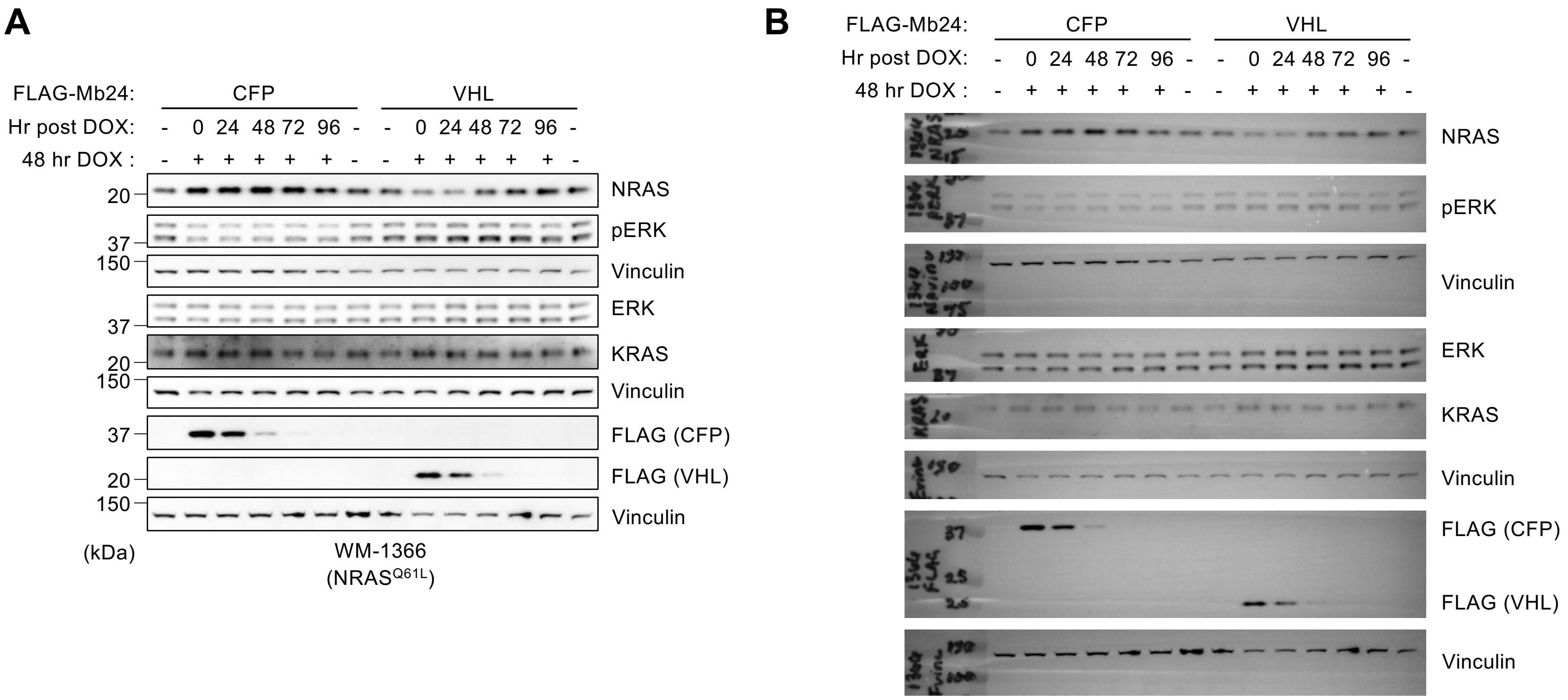

Supplemental Figure 16. Effect of NRAS degradation by VHL-Mb24 on NRAS-mediated signaling versus inhibition alone by CFP-Mb24 in WM-1366 cells. A) DOX was added for 48-hours and then removed from the media for indicated time points in WM-1366 cells stably expressing DOX-inducible CFP- or VHL-tagged Mb24. B) Raw data for Western Blot in Sup. Fig. 16A.

Supplemental Table 1

RAS Inhibitory Monobodies

Name	Platform	Isotype Specificity	Mutation Specificity	Nucleotide Specificity	Affinity (lowest K_D nM)	Epitope	PDB ID	Reference
**NS1**	Monobody	HRAS, KRAS	none	none	14	α4-β6-α5	5E 95	Spencer-Smith R, et al. (2017) Nat Chem Biol 13:62-68.
**12VC1**	Monobody	ND	G12C G12V	GTP	25	SI, SII, P-loop	7L0G	Teng KW, et al. (2021) Nat Commun 12:2656.
**R15**	Monobody	none	fast cycling	apo state	28	ND	-	Khan I, et al. (2022) Cell Rep 38:110322.
**JAM20**	Monobody	none	none	GDP	5	SI/SII pocket	-	Wallon et al (2022) PNAS, e2204481119.
**12D1, 2, 3, 4 & 5**	Monobody	KRAS	G12D	GTP/GDP	9.8	SII pocket	8EZG (12D1) 8F0M (12D5)	Akkepeddi et al (2023) PNAS, e2302485120.
**Mb24**	Monobody	NRAS	None	GDP	0.9	ND	-	This report

ND, not determined.
